# Laparoscopic Reduction and Closure of an Internal Hernia Secondary to Gynecologic Surgery

**DOI:** 10.1155/2017/5948962

**Published:** 2017-03-19

**Authors:** Takashi Sakamoto, Alan Kawarai Lefor

**Affiliations:** ^1^Department of Surgery, Tokyo Bay Urayasu Ichikawa Medical Center, 3-4-32 Todaijima, Urayasu, Chiba 279-0001, Japan; ^2^Department of Surgery, Jichi Medical University, 1-3311 Yakushiji, Shimotsuke, Tochigi 329-0498, Japan

## Abstract

Internal hernia is a rare cause of bowel obstruction which often requires emergent surgery. In general, the preoperative diagnosis of internal hernia is difficult. The pelvic cavity has various spaces with the potential to result in a hernia, especially in females. In this report, we describe a patient with an internal hernia secondary to previous gynecologic surgery. A 49-year-old woman presented with acute abdominal pain and a history of previous right oophorectomy for a benign ovarian cyst. Computed tomography scan of the abdomen showed obstruction with strangulation and emergent laparoscopic exploration was performed. Intraoperatively, there was an incarcerated internal hernia in the pelvis, located in the vesicouterine pouch, which was reduced. The orifice of the hernia was a 2 cm defect caused by adhesions between the uterus and bladder. The defect was closed with a continuous suture. The herniated bowel was viable, and the operation was completed without intestinal resection. She was discharged four days after surgery without complications. Laparoscopy is useful to diagnose bowel obstruction in selected patients and may also be used for definitive therapy. It is important to understand pelvic anatomy and consider an internal hernia of the pelvic cavity in females, in the differential diagnosis of bowel obstruction, especially those with a history of gynecological surgery.

## 1. Introduction

Intestinal obstruction is a common cause of abdominal distress resulting in patients seeking emergency care. Internal hernia is a rare cause of obstruction which may require emergent surgery and is defined as the extension of a viscus through a normal or abnormal orifice within the peritoneal cavity. Internal hernias have been reported to be responsible for 0.6–5.8% of cases of small bowel obstruction [1].

In general, the preoperative diagnosis of an internal hernia is difficult. Laparoscopic exploration may be useful for both diagnosis and treatment of a wide variety of abdominal conditions, in selected patients. The pelvis has spaces that may lead to herniation, especially in the female. In this report, we describe a patient with an internal hernia in the pelvis, secondary to previous gynecologic surgery with adhesion formation.

## 2. Case Presentation

**Figure 1:**
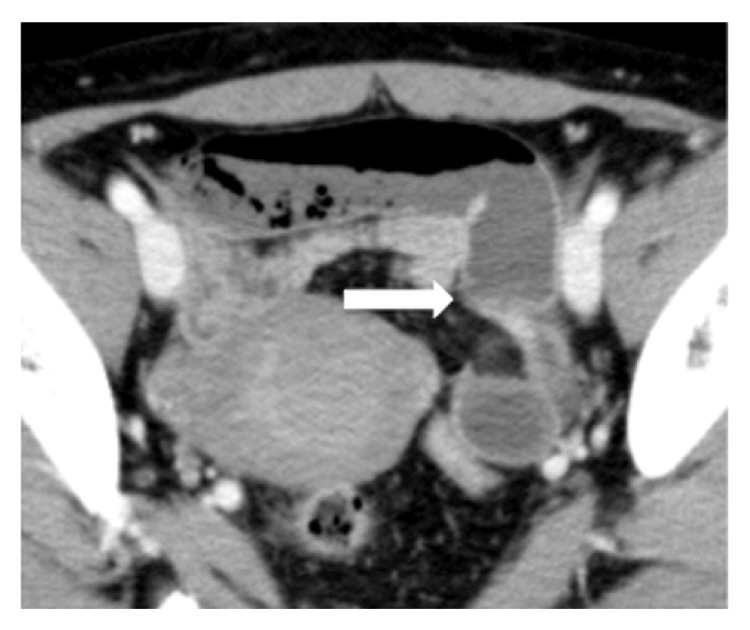
Abdominal computed tomography scan with intravenous contrast consistent with small bowel obstruction (axial view).* White arrow* shows the site of caliber change of the small intestine.

**Figure 2:**
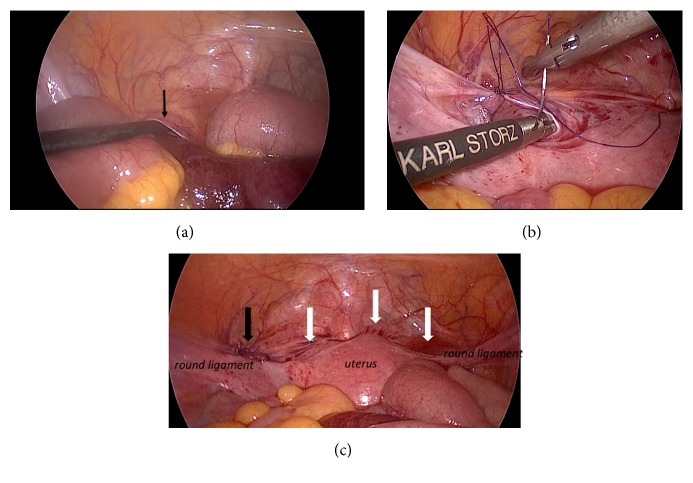
Intraoperative findings:* white arrows* show the pelvic adhesions,* black arrows* show the hernia orifice.

A 49-year-old female presented with acute abdominal pain. She has a history of open right unilateral oophorectomy for a benign ovarian cyst. She denied a history of chronic abdominal pain. On physical examination, she had severe tenderness in the left lower quadrant of the abdomen. Abdominal computed tomography (CT) scan was consistent with intestinal obstruction with strangulation (Figure 1). An internal hernia in the pelvis was suspected and emergent laparoscopic exploration was performed. Intraoperatively, the pelvis was clearly assessed and there was only a single adhesion, between the greater omentum and the abdominal wall. There was incarcerated small intestine passing through an internal hernia resulting from this adhesion in the left side of the pelvis, in the vesicouterine pouch, which was readily reduced. The orifice of the hernia was a 2 cm defect, which appeared to be secondary to a postoperative (postoophorectomy) adhesion between the uterus and bladder (Figure 2). The defect was closed with a continuous monofilament suture. The involved bowel was erythematous and congested but viable, and the operation completed without resection. She was discharged on the fourth postoperative day without complications.

## 3. Discussion

Internal hernia is defined as the protrusion of a viscus, most commonly small bowel, through a peritoneal or mesenteric orifice, resulting in its dislocation into another compartment [2]. Internal hernias represent only 0.6–5.8% of all small bowel obstructions [1]. Etiologically, internal hernias can be acquired or congenital. Acquired hernias are often due to mesenteric defects after bowel resection or changes in anatomy due to previous surgery such as a Roux-en-y bowel anastomosis.

Clinically, internal hernias can be asymptomatic or cause significant symptoms, which can include nausea, vomiting, or recurrent obstruction. Symptom severity relates to the duration and reducibility of the hernia and the existence of incarceration or strangulation [3]. In the past, these hernias were most frequently evaluated with small bowel oral contrast studies. Recently, abdominal CT scan has become the first-line imaging technique used in these patients. Radiographic features in barium studies include apparent encapsulation of distended loops of small intestine in an abnormal location, arrangement or crowding of small bowel loops within the hernia sac, and evidence of obstruction with segmental dilatation and stasis, with additional features of apparent fixation and reversed peristalsis during fluoroscopic evaluation. On CT scan, characteristic findings include mesenteric vessel abnormalities, with engorgement, crowding, twisting, or stretching of these vessels [3]. Even with advanced imaging modalities, identification of an internal hernia is often difficult, and surgical management is usually needed.

Pelvic adhesions secondary to gynecologic disease or surgery are common in adult females. There are several reports of internal hernias into the rectouterine pouch and vesicouterine pouch [4, 5]. There are many spaces in the pelvis which allow hernia sacs to develop, including the supravesical space, hernia through the broad ligament, vesicouterine pouch, pouch of Douglas (rectouterine), and perirectal pouch [6]. General surgeons may not be familiar with some gynecological diseases and may not consider the detailed anatomy of the pelvis. Most patients with intestinal obstruction, however, are seen by general surgeons. Surgeons must be very familiar with the spaces in the pelvis when managing patients with intestinal obstruction.

For diagnosis and surgical management, laparoscopic exploration of patients who may have an internal hernia is a worthwhile procedure. The location of adhesions after gynecological surgery may not hinder laparoscopic exploration. This would seem to be a prudent initial approach, while conversion to open surgery may be needed in some patients.

We present a patient with an internal hernia related to previous gynecologic surgery, treated with laparoscopic exploration and reduction. The operative findings emphasize the importance of understanding the anatomy of the pelvic space. Laparoscopic exploration is recommended in patients suspected to have intestinal obstruction with strangulation related to gynecologic disease.

## References

[1] Newsom B. D., Kukora J. S. (1986). Congenital and acquired internal hernias: unusual causes of small bowel obstruction. *The American Journal of Surgery*.

[2] Blachar A., Federle M. P. (2002). Internal hernia: an increasingly common cause of small bowel obstruction. *Seminars in Ultrasound CT and MRI*.

[3] Martin L. C., Merkle E. M., Thompson W. M. (2006). Review of internal hernias: radiographic and clinical findings. *American Journal of Roentgenology*.

[4] Bunni J., Teichmann D., Berstock J. R. (2012). Pouch of Douglas pelvic hernia: a rare entity managed laparoscopically. *Hernia*.

[5] Mou D., Seshadri A., Fallon M., Thummalapalli R., Askari R. (2016). Internal hernia through a congenital peritoneal defect in the vesico-uterine space. *International Journal of Surgery Case Reports*.

[6] Ma M. (2005). *Dynamic Radiology of the Abdomen: Normal and Pathologic Anatomy*.

